# New York Homœopathic Union

**Published:** 1893-12

**Authors:** E. C. D. O’Brien

**Affiliations:** Secretary, pro tem.


### NEW YORK HOMŒOPATHIC UNION.

The regular meeting of the Homœopathic Union took place at the residence of Dr. Edmund Carleton, 53 West Forty-fifth Street, New York, on the evening of October 19th, the President, Dr. Carleton, in the chair. There were present Drs. Allan, Baylies, Burn, Carleton, Dyer, Finch, Fincke, W. M. James, of Philadelphia; Thomson and O'Brien. In the absence of the Secretary, Dr. Stanton, Dr. O'Brien took the minutes of the meeting. Sections 190 to 200 inclusive of *The Organon* were read and discussed. These sections relate to the treatment of disease by the internal remedy alone and not in conjunction with local applications.

### DISCUSSION.

Dr. Carleton—I regard this as one of the most important parts of *The Organon.* Many professed homœopathic physicians violate this law.

Dr. Allan—Ophthalmia neonatornm can be cured better with internal medication than with local applications. It is not necessary to use Atrophine in iritis, the indicated remedy being sufficient; but if the inexperienced physician is uncertain of the remedy and delays too long, the iris will be fastened to the lens, and in such a case the use of Atropine will dilate the pupil and prevent adhesions.

Dr. Carleton—Is it more difficult to prescribe for the eye than for other parts of the body?

Dr. Thomson—Had experienced no difficulty with children suffering with diseased eyes, and had seen many cases injured by external applications.

Dr. Baylies—Had treated conjunctivitis successfully with the internal remedy alone.

Dr. Allan—Does not approve of local applications.

Dr. Thomson—Cited a case of pin-head pupil where remedies had been given and local applications used by another physician. He gave Physostigma and in a quarter of an hour relief was obtained. Did not know if case was traumatic.

Dr. Baylies—Had a case of tetanus and trismus in which Physostigma helped. Angostura cured the tetanus.

Dr. James—Said the use of Atropine locally was parrot work. It produces congestion of the eye and may cause hemorrhage of the retina. A boy was struck in the eye by a stone, causing temporary blindness. Atropine was introduced and the retina destroyed.

Dr. Carleton—The trouble was traumatic; and that led the doctors to resort to the surgical methods of the old school, losing sight altogether of the benefits to be derived from careful homœopathic prescribing.

Dr. James—Mentioned a case of glaucoma where Atropine was used, and patient was afterward cured by Argent-nit., given internally, by a true homœopath.

Dr. Allen—Belladonna aggravates glaucoma, causing hemorrhage into the retina and the optic nerve is destroyed by pressure. A case of glaucoma of right eye was cured by Bell.$^{cm}$. A diarrhœa resulted and another physician gave two remedies in alternation: the glaucoma returned and the eye had to be removed.

Dr. James—The diarrhœa should have been let alone.

Dr. Allan—Yes; it might have subsided the next day.

Dr. James—Dr. Lippe was called in consultation to a very serious case. The remedy he gave worked grandly, but uterine hemorrhage supervened. The doctor in attendance thought something should be done for it, and used mechanical measures which stopped the bleeding at once, and the patient died within twenty-four hours after that. Dr. Lippe said the hemorrhage would have stopped by itself.

Dr. Carleton—What has been the experience of members with epistaxis in this connection?

Dr. Thomson—Had a patient, a man seventy-two years old, with persistent hemorrhage; gave remedy every half-hour and cured the case.

Dr. Finch—Had a severe case of epistaxis, patient nearly exsanguinated. Injected Tannic-acid, patient recovered, but doctor did not feel proud of cure as he grew wiser.

Dr. Fincke—Cured a case of epistaxis in a few minutes with Acon.$^{cm}$. Cured a case with China$^{50m}$; to satisfy family, promised to resort to surgical means within eight minutes if hemorrhage did not lessen; it ceased within the time. The physician often loses courage through anxiety and fear. Many women are sacrificed in obstetrical work through fear. We should study remedies.

Dr. Thomson—Had a patient whose menstruation ceased while at the theatre. Epistaxis came on and the patient came to him in a fright. He gave Placebo, as she was under a remedy.

Dr. Fincke—Has any one had a case of epistaxis where blood was cold? He could not find any remedy with that symptom.

Dr. James—What remedy has profuse cold saliva?

Dr. Thomson—Was Dr. Fincke's epistaxis cold to touch?

Dr. Fincke—Answered yes. He then referred to orificial surgery which has come among us. He said: The paragraphs under discussion concerning the treatment of so-called local diseases are intimately connected with the vital point of homœopathics, the sufficiency of homœopathic treatment, and prove it beyond doubt. It is on that account mainly that the majority of homœopaths are switching off to innumerable adjuvants and surgery in cases which are amenable to Homœopathy and therefore should be treated accordingly. That surgery itself is only a necessary adjunct and has by no means the wide range attributed to it in modern times, Hahnemann has distinctly asserted in § 186, in the words: surgical treatment is indicated "where a mechanical aid is to be applied to the suffering parts, so that the external impediments to the healing, to be expected through the life-force alone, be annihilated." This limit should constantly be borne in mind by every homœopathician. Many have no idea how far the homœopathic remedy reaches, if properly applied in simility and potency. But for the purpose of obtaining all the good things it is able to accomplish we must let it do its own work without disturbing it by our anxiety to benefit the case and rushing into a multitude of remedies, contrivances, and auxiliaries which throw the life-force in confusion and by its waste diminish its resources instead of increasing them by calm judgment. It wants nerve to do as Hahnemann desires, but the result rewards our earnest efforts in the relief and cure of our patients, and that is all the sufficiency needed in therapeutics.

Dr. Carleton—Retribution will follow soon. The returns are beginning to come in. I took pains to keep informed of three cases where the "American operation" had been performed for some trouble of the rectum. The principal part of the operation consists in removing one inch of rectum. The patients seemed better for a short time, but now are worse than ever.

Dr. Baylies—Progressive muscular atrophy is treated by stretching the sphincter.

Dr. James—Mentioned a case of typhoid where, under old-school treatment, the blood of a goat was used for transfusion, and patient died.

Dr. Fincke—Thought local application for disease would neutralize internal remedy.

Dr. Thomson—It appears to heal from outside, but should be from within.

Dr. Finch—If the remedy be given for external trouble, and something new results, such as diarrhœa, should the case be then left to nature or should another remedy be given?

Dr. Thomson—Where the indicated remedy had been given let the case alone. In traumatic troubles, such as fractures, set the bones, place them in splints, and use such external applications as are necessary. Then study the case as a physician.

Dr. Baylies—The too frequent use of a remedy will retard the healing of a fracture.

Dr. Finch—If a chronic disease disappears under treatment and something else takes its place, will a new remedy cure the new disease and cause the old one to return?

Dr. Carleton—If the case is better and new symptoms appear do not give a remedy.

Dr. Finch—Had a patient—a woman—with a bad cough. Prescribed two or three times with trifling result; then traced the history of the case backward to puberty, and found she had at that time been cured by a professed homœopathist of a persistent leucorrhœa, an itching eruption, and constipation. One lung now affected. Carefully selected remedy, the eruption first returned, in a few days the constipation came back, and finally the leucorrhœa. All disappeared in the order of their coming without any new remedy.

Dr. Thomson—Looks like the action of an anti-psoric remedy, yet as one remedy cured does not look like a psoric case.

Dr. Finch—The cough improved.

The meeting then adjourned.

E. C. D. O'Brien, M. D.,
*Secretary, pro tem.*

---

